# High Prevalence of Fecal Carriage of Extended Spectrum β-Lactamase-Producing Enterobacteriaceae Among Newly HIV-Diagnosed Adults in a Community Setting in Tanzania

**DOI:** 10.1089/mdr.2020.0066

**Published:** 2020-12-03

**Authors:** Joel Manyahi, Sabrina John Moyo, Marit Gjerde Tellevik, Nina Langeland, Bjørn Blomberg

**Affiliations:** ^1^ Department of Clinical Science, University of Bergen, Bergen, Norway.; ^2^ National Advisory Unit on Tropical Infectious Diseases, Haukeland University Hospital, Bergen, Norway.; ^3^ Department of Microbiology and Immunology, Muhimbili University of Health and Allied Sciences, Dar es Salaam, Tanzania.

**Keywords:** ESBL, HIV, community, Tanzania

## Abstract

Colonization in HIV-infected populations with extended-spectrum β-lactamase-producing Enterobacteriaceae (ESBL-PE) is particularly worrisome in low-income settings. This study describes the prevalence of ESBL-PE carriage and associated risk factors among newly HIV-diagnosed adults in a community setting in Tanzania. A total of 595 newly diagnosed HIV adults with a median age of 35 years with interquartile range (IQR) 29–42 years and a median CD4 count of 492 cells/μL (IQR 390–666 cells/μL) were recruited. Among these, 194/595 (32.6%, 95% confidence interval [CI] 28.9–36.6) were ESBL-PE carriers. Participants with low CD4 count (<350 cells/μL) had significantly higher prevalence of ESBL-PE carriage compared with those with CD4 count ≥350 cells/μL (26/58, 44.8%, vs. 168/537, 31.3%, *p* = 0.04). Antibiotic use in last 4 weeks (odds ratio [OR] 1.55, 95% CI 1.08–2.22, *p* = 0.02) and CD4 count ≥350 cells/μL (OR 1.78, 95% CI 1.03–3.09, *p* = 0.04) were independent risk factors for fecal carriage of ESBL-PE. In total, 244 isolates of ESBL-PE were isolated from 194 participants. Of these, 238/244 (97.5%) harbored *bla*_CTX-M_ genes, with *bla*_CTX-M-15_ being predominant (219/238 (92%), followed by *bla*_CTX-M-27_ (9/238 (3.8%), *bla*_CTX-M-14_ (8/238 (3.4%), *bla*_CTX-M-55_ (1/238), and *bla*_CTX-M 211/3_ (1/238). *bla*_SHV-2a_ genes were detected in four isolates, whereas the *bla*_SHV-12_ gene was detected in one isolate. Phenotypic carbapenemase-producing Enterobacteriaceae was detected in one HIV-positive person with CD4 count 132 cells/μL. In conclusion prevalence of ESBL-PE carriage is high among newly diagnosed HIV adults in Dar es Salaam, and is significantly associated antibiotic use and low CD4 count.

## Introduction

Individuals living with HIV are at risk not only of classical HIV-related opportunistic infections such as pneumocystis pneumonia and tuberculosis, but also severe infections caused by common bacterial pathogens such as *Escherichia coli*, salmonella, pneumococci, and staphylococci.^1–3^ The World Health Organization (WHO) recognizes antimicrobial resistance (AMR) in such bacterial infections as a major threat to global health. Extended-spectrum β-lactamase-producing Enterobacteriaceae (ESBL-PE) constitute a particular challenge, as almost all are multidrug resistant. Previously, infections due to ESBL producers were mainly a health care-associated problem.^4,5^ However, ESBL-PE infections are now increasingly acquired in the community.^6,7^

In resource-constrained settings, ESBL-PE infections are spread more easily due to deficient infection control, whereas expensive reserve antibiotics such as carbapenems and colistin are inaccessible. Consequently, infections caused by ESBL-PE carries high mortality exceeding mortality outcome of bacterial infections in the preantibiotic era.^5,8^

Colonization with ESBL-PE frequently precedes invasive diseases.^9,10^ Fecal carriage of ESBL-PE could pose greater risk in people living with HIV due to the increased risk of severe bacterial infections.^1,2^ In Tanzania, studies have documented high frequency of infections caused by ESBL-PE in hospitalized patients.^5,11–13^ ESBL-PE carriage is also common in hospital settings, and carriage rates of 50% have been documented in hospitalized children in Tanzania.^14^ Even in community settings, studies have documented ESBL-PE carriage rates of 11–32% in healthy children^15^ and 17% in healthy adults^16^ in the same country. Although high rates (89.7%) of ESBL-PE carriage have been reported in hospitalized HIV positive children in Tanzania,^14^ others documented lower rates (14%) in HIV-infected children in the community in Zimbabwe.^17^ To date, no community-based studies from Africa have reported frequencies and risk factors for ESBL-PE carriage among newly diagnosed HIV adults.

HIV is pandemic in sub-Saharan Africa, and Tanzania has an estimated 1.4 million people aged between 15 and 64 years living with HIV.^18^ We designed this study aimed to determine prevalence of fecal carriage of ESBL producers in HIV-infected people in the community; and assess any influence of antibiotic use, hospitalization, and CD4 count on fecal carriage of ESBL producers. The findings may help develop strategies for reducing the burden of AMR in a population at high risk of severe infections.

## Materials and Methods

### Study sites and study participants

Newly diagnosed HIV-infected adults with CD4 count of ≥350 cells/μL who had not yet started antiretroviral treatment (ART) were recruited consecutively as part of a double-blinded randomized clinical trial (RCT), CoTrimResist (ClinicalTrials.gov identifier: NCT03087890) between April 2017 and May 2018. The study was performed in Dar es Salaam, Tanzania, the largest city and economic capital of Tanzania. Participants were residents of the main municipals of Dar es Salaam city, namely Kinondoni, Temeke, Ilala, Ubungo, and Kigamboni, and were recruited through six HIV care and treatment clinics at Mwananyamala Hospital, Amana Hospital, Temeke Hospital, Pastoral Activities and Services for People with AIDS Dar es Salaam Archdiocese (PASADA), Mbagala Hospital, and Mnazimmoja Hospital. In addition, newly diagnosed HIV-infected adults with CD4 counts of <350 cells/μL (*n* = 58) were recruited from the same Hospitals during screening for eligibility to the RCT. This article reports on the baseline data before interventions and treatment.

### Data collection

Health workers recorded participants' data such as demographics and clinical parameters on tablet computers using standardized electronic case report forms employing the RedCAP system (Research Electronic Data Capture, Vanderbilt University, Nashville, TN), which automatically synchronizes study data to a central data server.

### Screening for ESBL- and carbapenemase-producing Enterobacteriaceae

From each participant, a rectal swab was collected and transported in liquid Cary-Blair medium (Fecal Transwab, MWE Co Bath Ltd., Corsham, United Kingdom) in a cool box and maintained at 4°C with icepack. Rectal swabs were inoculated into brain heart infusion (BHI) broth and incubated overnight at 37°C. Two drops (0.1 mL) from BHI broth were subcultured on CHROMID ESBL (BioMérieux, Marcy l'Etoile, France) for screening of ESBL-PE,^19^ and CHROMID CARBA SMART (BioMérieux) for combined screening for carbapenemase-producing Enterobacteriaceae (CPE) and OXA-48-producing Enterobacteriaceae. The plates were incubated at 37°C for 24 hours and bacterial growth interpreted according to manufacturer's instructions. Molecular characterized strains harboring different combinations of β-lactamases were used for quality control as previously described.^14^

Bacterial isolates were identified by MALDI-TOF MS using the Microflex LT instrument and MALDI Biotyper 3.1 software (Bruker Daltonics, Bremen, Germany).

### PCR and sequencing for detection and identification of ESBL genes

DNA was extracted by a rapid boiling procedure and stored at −20°C. Real-time PCR was used for detection of cefotaxime-Munich (CTX-M) encoding genes using a LightCycler 480 Instrument II (Roche Diagnostics, Mannheim, Germany). Forward primer CTXM-F 5′-ATGTGCAGYACCAGTAARGT-3′, and reverse primers CTXM-R1 5′-TGGGTGAAGTAAGTGACCAGA-3′ and CTXM-R2 5′-TGGGTAAARTAGGTCACCAGA-3′ (TIB Molbiol, Berlin, Germany), which target a 595 bp internal region present in all the five different CTX-M groups, were used as previously described.^14^ Each run included positive and negative controls in duplicates. The PCR products were sequenced using the same reverse primers as for PCR. Sequencing was done using BigDye Terminator v1.1 Cycle Sequencing Kit (Applied Biosystems, Foster City, CA). Sequences were analyzed using the Basic Local Alignment Search Tool program available at the website of the National Center for Biotechnology Information.

For confirmation of ESBL producers, isolates negative for CTX-M PCR were tested for the presence of *bla*_SHV_ genes as previously described by Kommedal *et al.*^20^

### Statistical analysis

We used chi-square test to assess the proportions of fecal carriage of ESBL producers by patient characteristics. We analyzed the association between risk factors of interest (antibiotic use, prior hospitalization, and low CD4 counts) and fecal carriage of ESBL producers, adjusting for age, gender, education status, district of residence, and study sites (individual study sites). For each main exposure variable, we assessed all potential confounders using both logistic regression and Mantel–Haenszel method, defining a confounder as a factor that changes the effect size by ≥10%. Effect modification of factor between different groups were examined by Mantel–Haenszel method. A significance level of 0.05 was used and all *p*-values refer to two-sided tests. Statistical analysis was performed using STATA version 16 (College Station, TX).

### Ethical approval and consent to participate

Ethical approval to conduct the study in Tanzania was obtained from Muhimbili University of Heath and Allied Sciences senate research and publication committee (Ref. No. 2015-10-27/Vol.X/54)—Muhimbili University of Health and Allied Sciences, National Institute for Medical Research (NIMR/HQ/R.8a/Vol.IX/2144)—Tanzania, Ministry of Health, Community development, Gender, Elderly, and Children. The clinical trial was also registered by Tanzania Food and Drugs Authority (TZ16CT007) and Clinical trial.org (NCT03087890). In Norway, the study was approved by the Regional Committee for Medical and Health Research Ethics of Western Norway (REK2015/540). Informed written consent was obtained from each of the study participants.

## Results

### Description of study participants

We recruited a total of 595 newly HIV-diagnosed adults with a median age of 35 years with interquartile range (IQR) 29–42 years and a median CD4 count of 492 cells/μL with IQR 390–666 cells/μL. Among these, 537 (90.2%) were newly diagnosed HIV seropositive individuals with CD4 counts ≥350 cells/μL (median 518 IQR 413–687) and 58 (9.8%) were newly diagnosed HIV seropositive individuals with CD4 counts of <350 cells/μL (median 159 IQR 61–259). Out of 595, 451 (75.8%) were female and 233 (39.2%) were aged between 30 and 39 years. The majority, 360/595 (60.5%), of the HIV seropositive individuals had WHO stage 1 HIV disease. Although 64/595 (10.8%) of participants had been admitted to hospital within the last year, 191/595 (31.7%) reported use of antibiotics during the last 4 weeks before enrollment (Table 1).

**Table 1.** (Clinical Demographics) Characteristics of the Study Participants

Variable	Frequency	Percentage
Gender		
Female	451	75.8
Male	144	24.2
Age (years)		
Up to 29	165	27.7
30–39	233	39.2
40–49	148	24.9
50–59	33	5.5
60 and above	16	2.7
Education		
Primary school	388	65.2
Secondary	126	21.2
Postsecondary	24	4.0
None/informal	57	9.6
District of residence		
Temeke	289	48.6
Kinondoni	114	19.1
Ubungo	32	5.4
Ilala	139	23.4
Kigamboni	12	2.0
Others	9	1.5
HIV stage		
1	360	60.5
2	173	29.1
3	59	9.9
4	3	0.5
CD4 counts (cells/μL)		
≥350	537	90.2
<350	58	9.8
Study site		
PASADA	168	28.2
Temeke	54	9.1
Amana	30	5.0
Mwananyamala	100	16.8
Mbagala	124	20.9
Mnazimmoja	119	20.0
Hospital admission within last year		
No	531	89.2
Yes	64	10.8
Antibiotic use last 4 weeks		
No	404	67.9
Yes	191	32.1

PASADA, Pastoral Activities and Services for People with AIDS Dar es Salaam Archdiocese.

### Prevalence of ESBL carriage and identification of the bacterial isolates

The overall prevalence of fecal carriage of ESBL-PE was 194/595 (32.6%, 95% confidence interval [CI] 28.9–36.6), and the proportions of carriage in subgroups are shown in Table 2. The prevalence of fecal carriage of ESBL-PE varied between study sites (Table 2). Participants with low CD4 counts (<350 cells/μL) had significantly higher prevalence of ESBL-PE carriage than those with higher CD4 counts (44.8%, 26/58 vs. 31.3%, 168/537, *p* = 0.04).

**Table 2.** Prevalence of Extended-Spectrum β-Lactamase-Producing Bacteria by Clinical-Demographic Characteristics

Variable	Frequency	Prevalence of ESBL, % (*n*)	Prevalence of ESBL, % (*n*)
Gender			
Female	451	32.6 (147)	
Male	144	32.6 (47)	1.000
Age (years)			
Up to 29	165	35.8 (59)	
30–39	233	30.0 (70)	
40–49	148	32.8 (50)	
50–59	33	18.2 (6)	
60 and above	16	56.3 (9)	0.068
Education			
Primary school	388	30.2 (117)	
Secondary	126	31.7 (40)	
Postsecondary	24	50.0 (12)	
None/informal	57	43.9 (25)	0.053
District of residence			
Temeke	289	29.4 (85)	
Kinondoni	114	38.6 (44)	
Ubungo	32	34.4 (11)	
Ilala	139	36.7 (51)	
Kigamboni	12	0.0 (0)	
Others	9	33.3 (3)	0.072
HIV stage			
1	360	31.9 (115)	
2	173	31.8 (55)	
3	59	40.7 (24)	
4	3	0.0 (0)	0.344
CD4 counts (cells/μL)			
≥350	537	31.3 (168)	
<350	58	44.8 (26)	0.037^*^
Study site			
PASADA	168	22.0 (37)	
Temeke	54	20.4 (11)	
Amana	30	33.3 (10)	
Mwananyamala	100	40.0 (40)	
Mbagala	124	39.5 (49)	
Mnazimmoja	119	39.5 (47)	0.001^*^
Hospital admission within last year			
No	531	33.1 (176)	
Yes	64	28.1 (18)	0.418
Antibiotic use last 4 weeks			
No	404	29.5 (119)	
Yes	191	39.3 (75)	0.017^*^

^*^ *p*-Values calculated by chi-square test with 0.05 as cutoff for statistical significance.

ESBL, extended-spectrum β-lactamase.

A total of 244 isolates of Enterobacteriaceae were isolated from 194 participants harboring ESBL-PE phenotypes. The majority of participants, 74.2%, were colonized by a single ESBL-PE isolate, whereas 50/194 (25.8%) had two or more bacterial isolates. *E. coli* was the predominant microbe, 209/244 (85.7%), followed by *Klebsiella pneumoniae*, 33/244 (13.5%), and *Enterobacter cloacae*, 2/244 (0.8%). Patients with CD4 counts <350 cells/μL were more likely to carry multiple isolates of ESBL-PE than those with higher CD4 counts (15.5%, 9/58 vs. 6.9%, 37/537, *p* = 0.02). One *E. coli* isolate was CPE and isolated from an HIV participant with CD4 count 132 cells/μL.

### ESBL genotypes

PCR confirmed ESBL genotype for 242/244 (99.2%) of isolates with phenotypic ESBL-PE. The majority, 238 (97.5%), harbored *bla*_CTX-M_ encoding genes. The most predominant *bla*_CTX-M_ gene was *bla*_CTX-M-15_ (219/238, 92%), followed by *bla*_CTX-M-27_ 9/238 (3.8%), *bla*_CTX-M-14_ 8/238 (3.4%), *bla*_CTX-M-55_ (1/238), and *bla*_CTX-M 211/3_ (1/238). All *K. pneumoniae* and *E. cloacae* isolates carried the *bla*_CTX-M-15_ gene only, whereas *E. coli* displayed a diversity of *bla*_CTX-M_ genes, including the ones mentioned earlier. *Bla*_SHV-2a_ genes were detected in two *E. coli* and two *K. pneumonia*, whereas the *bla*_SHV-12_ gene was detected in one *E. coli*.

### Predictors of ESBL producers

Among the main risk factors of interest, only antibiotic use during the last 4 weeks (odds ratio [OR] 1.55, 95% CI 1.08–2.22, *p* = 0.02) and CD4 count <350 cells/μL (OR 1.78, 95% CI 1.03–3.09, *p* = 0.04) were independently associated with fecal carriage of ESBL producers (Table 3). Prior hospitalization during the last 1 year was not a significant risk factor (OR 0.79, 95% CI 0.44–1.40, *p* = 0.41). The effect size of each risk factors did not change significantly (>10%), whereas adjusting for the other main risk factors and potential confounders and effect modifiers, that is, age, gender, education status, district of residence, and study sites (individual study site). Thus, we did not find any significant confounders or effect modifiers.

**Table 3.** Risk Factors for Fecal Carriage of Extended-Spectrum β-Lactamase Producers in HIV Patients

Variable	Frequency	ESBL carriage, % (*n*)	OR	95% CI	*p*
CD4 counts (cells/μL)^*^					
≥350	537	31.3 (168)	1		
<350	58	44.8 (26)	1.78	1.03–3.09	0.04
Antibiotic use last 4 weeks^†^					
No	404	29.5 (119)			
Yes	191	39.3 (75)	1.55	1.08–2.22	0.02
Hospital admission within last year					
No	531	33.1 (176)	1		
Yes	64	28.1 (18)	0.79	0.44–1.40	0.41

^*^ Adjusted for antibiotic use in last 4 weeks, study sites, hospital admission with last year, age, gender, and education status.

† Adjusting for age, gender, education status, hospital admission within last year, CD4 count and study sites.

CI, confidence interval; OR, odds ratio.

## Discussion

This first community-based study reports prevalence of fecal carriage of ESBL-PE in almost one third (194/595, 32.6%) of individuals newly diagnosed with HIV in Dar es Salaam. This rate of ESBL-PE fecal carriage is much higher than found among adults with unknown HIV status in community-based studies in Northern Tanzania (55/334, 16.5%, *p* < 0.001),^16^ Burkina Faso (22/101, 21.8%, *p* = 0.03),^21^ Gambia (28/565, 5.0%, *p* < 0.001),^22^ and Morocco (4/93, 4.3%, *p* < 0.001).^23^ Although we are not aware of other community-based studies from HIV-infected adults in Africa, there was significantly lower ESBL-PE carriage rate (24/175 13.7%, *p* < 0.001) in a community-based studies of HIV-infected children in Zimbabwe.^17^ The high carriage rate observed in our study could be attributed to several factors, including the choice of screening technique employed. Our use of broth enrichment of rectal swabs may have increased the yield in detecting ESBL-PE colonization.^24,25^ In contrast, our use of rectal swabs may have inferior sensitivity compared with stool culture used in other studies.^19^

We observed that antibiotic use during the last 4 weeks was a strong risk factor for fecal carriage of ESBL producers in this cohort. In Tanzania, almost all antibiotics can be accessed over the counter. Overuse of antibiotics and misuse of broad-spectrum antibiotics, such as cephalosporins for trivial infections, are known to contribute to increasing AMR, including ESBL-PE. In this setting, people with an undiagnosed HIV infection are likely to opt for self-medication with antibiotics for intercurrent infections and symptoms related to an, as yet, unrecognized immunodeficiency. Such overuse of antibiotics may partially explain the high prevalence of ESBL-PE carriage in our study. This study confirms our hypothesis that antibiotic use affects carriage of resistant bacteria in HIV populations, and our finding is consistent with earlier observations from community and hospital settings in Africa.^14,16,21,22^

Previous hospitalization is a well-known risk factor for fecal carriage of ESBL producers,^12,21^ contributing both to increased likelihood of antibiotics uses and acquisition of ESBL producers in gastrointestinal tract. However, this study could not add support this observation. Tanzania's current implementation of the “test and treat” strategy for HIV is expected to result in more people getting diagnosed before actually experiencing symptomatic disease. Despite this, it is plausible that many patients will have consulted health care services, without actually having been admitted, for unknown HIV-related ailments before getting diagnosed with HIV, and that this may have led to increased risks of gastrointestinal colonization with ESBL-PE.

This study found low CD4 count (<350 cells/μL) was an independent risk factor for fecal carriage of ESBL. Low immunity predisposes to risk of opportunistic infections increasing the likelihood of antimicrobial use and hospitalization. However, adjustment for both antibiotic use and hospitalization did not alter the independent association between low CD count and fecal carriage of ESBL producers. Furthermore, Wilmore et al.^17^ found no association between low CD count and fecal carriage of ESBL producers in children in Zimbabwe.^17^ The differences could be explained by inclusion criteria, this study recruited newly diagnosed HIV-infected adults not yet on ART, whereas Wilmore *et al.* enrolled children who were stable on ART.^17^

Our study confirmed *bla*_CTX-M 15_ as the predominant ESBL gene type accounting 92% of all isolates, supporting other reports on the extraordinary dissemination of the *bla*_CTX-M-15_ genotype worldwide. In the first ever report of ESBL-PE from Tanzania,^5,13^ we showed that *bla*_CTX-M-15_, alongside TEM-63, was the dominant cause of ESBL-PE bloodstream infections in Tanzania as early as 2001–2002. Later on, we found predominance of *bla*_CTX-M-15_ in clinical isolates from community-acquired urinary tract infections from 2004 (Ref.^7^). Reports from elsewhere^26,27^ indicate that when *bla*_CTX-M-15_ genotype penetrates in a new landscape where other ESBL genes persist, *bla*_CTX-M-15_ tend to displace other genes and becomes predominant. All recent studies from community and hospital settings in Tanzania have supported the predominance of *bla*_CTX-M 15_ genes among Enterobacteriaceae isolated from feces.^12,15,16^ The CTX-M group 9, *bla*_CTX-M-27_, and *bla*_CTX-14_ were also documented in this study and were only isolated from *E. coli*. This correlates with a recent study among HIV-infected children in Zimbabwe.^17^ Our previous study on uropathogenic isolates did not detect any *bla*_CTX-M_ group 9 genes,^7^ whereas a pediatric study reported only two *E. coli* isolates carrying *bla*_CTX-M-14_ genes and none with *bla*_CTX-M-27_.^14^ In conclusion, the study found a high prevalence of fecal ESBL-PE carriage among people newly diagnosed with HIV. Antibiotic use during the last 4 weeks and low CD4 count were risk factors for fecal carriage of ESBL producers. Further studies should investigate transmission dynamics of ESBL-PE in the community, complex factors driving the emergence of multidrug-resistant bacteria, and the relationship between ESBL-PE carriage and invasive disease. Social behavior and policies on AMR need to be crafted and regulation on antimicrobial use must be enforced.
